# 2017 Riley Heart Center Symposium on Cardiac Development: Development and Repair of the Ventricular Wall

**DOI:** 10.1007/s00246-018-1942-4

**Published:** 2018-07-31

**Authors:** Loren J. Field, Weinian Shou, Larry Markham

**Affiliations:** The Riley Heart Center and the Herman B Wells Center for Pediatric Research, Indiana University School of Medicine, Indianapolis, IN USA

Abnormal heart morphogenesis can result in congenital heart defects (CHDs) or inherited cardiomyopathies. Inappropriate gene expression [1, 2], expression of mutant gene products [3, 4], and exposure to cardiotoxic chemicals [5] or drugs [6] are all known to promote CHDs. The resulting structural defects (exemplified by heterotaxy, atrial and ventricular septal defects, noncompaction, etc.) render heart pump function inadequate. Nearly 1% of all newborns will have a structural heart defect [7], and the majority of these are severe enough to cause death in the absence of surgical and/or other palliative intervention. Inherited cardiomyopathies (that is, abnormalities of the sarcomere) [8, 9] constitute another important class of CHDs.

While much is known about the clinical sequelae of CHDs, in many cases, the underlying molecular etiology remains undefined. For example, visceral heterotaxy results from the loss of left–right patterning during early embryogenesis, when the cell and molecular signaling cascades, which normally regulate sidedness pattern formation, are well-established. However, the spectrum of cardiac defects in many heterotaxy models and within patients with heterotaxy is more severe than what would be anticipated from a simple breakdown of sidedness patterning. The molecular basis for this is not understood. Similarly, the anatomical and clinical sequelae resulting from anomalies in ventricular septation and papillary muscle morphogenesis are well characterized. Moreover, multiple genes have been identified, which when dysregulated impact the development of these structures. How dysregulated gene expression mechanistically gives rise to these morphogenic defects is at best poorly understood. A similar case can be made for events that regulate maturation of the ventricular wall. Left ventricular noncompaction (LVNC) is recognized as a distinct form of cardiomyopathy, which results from a morphogenic defect. However, the molecular processes that underlie this defect are not well understood; understanding the underlying molecular mechanisms which give rise to LVNC is critical to effect improvements in diagnosis and care [10].

To promote interactions between clinical and basic scientists working on CHDs, the Riley Heart Center at Riley Hospital for Children, Indiana University School of Medicine, has organized a series of symposia focusing on various aspects of cardiac development. Previous symposia were entitled “Growth and Morphogenesis of the Ventricular Wall” (2008), “Transcriptional Unification of Heart Morphogenesis” (2009), “Cardiomyocyte Injury and Protection” (2010), and “Development of the Cardiac Conduction System and Arrhythmias” (2011). The proceedings of these symposia were published in special issues of Pediatric Cardiology. Several articles reviewing the topics discussed at the 5th Riley Heart Center Symposium (2017), entitled “Development and Repair of the Ventricle Wall,” are published in the prior and this issue of *Pediatric Cardiology*. The 5th Riley Heart Center Symposium was held in Indianapolis, Indiana, on November 20, 2017.

The symposium faculty included 14 leading clinical and basic scientists from the United States, Germany, and The Netherlands. Sessions at the symposium focused on (1) the genetic regulation of cardiac development, (2) the role of cell polarity in ventricular wall morphogenesis, (3) the cell and molecular basis for arrhythmic cardiomyopathies, and (4) cell cycle-based interventions to promote myocardial regeneration. The articles provide a state of the art review of basic and clinical studies focused on the regulation and evaluation of ventricular wall growth in experimental animals and in man. It is hoped that these proceedings, as well as those from future Riley Heart Center Symposia, will foster collaborative projects between clinical and basic researchers who are dedicated to improving the treatment, and ultimately preventing the onset, of heart failure in the young.
